# Nano-carriers of COVID-19 vaccines: the main pillars of efficacy

**DOI:** 10.2217/nnm-2021-0250

**Published:** 2021-10-11

**Authors:** Carolina Constantin, Anissa Pisani, Giuseppe Bardi, Monica Neagu

**Affiliations:** ^1^“Victor Babeş” National Institute of Pathology, 99-101 Spl Independentei, Bucharest, 050096, Romania; ^2^Colentina Clinical Hospital, 19-21, Sos. Stefan cel Mare, Bucharest, Romania; ^3^Nanobiointeractions & Nanodiagnostics, Istituto Italiano di Tecnologia, Via Morego 30, Genova, 16163, Italy; ^4^Department of Chemistry & Industrial Chemistry, University of Genova, Via Dodecaneso 31, Genova, 16146, Italy; ^5^University of Bucharest, 93–95 Spl Independentei, Bucharest, Romania

**Keywords:** COVID-19, immunity, nanoparticles, SARS-CoV-2, vaccine carrier, vaccines

## Abstract

As the current COVID-19 pandemic illustrates, vaccination is the most powerful method of disease prevention and public confidence in vaccines depends on their safety and efficacy. The information gathered in the current pandemic is growing at an accelerated pace. Both the key vital protein DNA/RNA messengers and the delivery carriers are the elements of a puzzle including their interactions with the immune system to suppress SARS-CoV-2 infection. A new nano-era is beginning in the vaccine development field and an array of side applications for diagnostic and antiviral tools will likely emerge. This review focuses on the evolution of vaccine carriers up to COVID-19-aimed nanoparticles and the immune-related adverse effects imposed by these nanocarriers.

COVID-19, induced by SARS-CoV-2, has caused an extended global pandemic. Various means have been applied to combat this pandemic, such as novel drugs, new therapeutic approaches for difficult cases, as well as new outlines for clinical management [1,2]. However, among the various modalities that could halt this pandemic, including hypothesized nonconventional approaches using nanomaterials [3], the achievement of herd immunity is the optimal solution. Herd immunity, also known as community immunity, is reached when a large amount of the population within a community becomes immune to a specific disease (whether naturally or artificially immunized) ending the infectious agent spread [4].

The optimal way to obtain preventive herd immunity before a viral infection is the development of efficient and safe vaccines. For the current pandemic, a broad international vaccination campaign including low-income countries should proceed as quickly as possible. In July 2020, the SARS-CoV-2 panel of vaccines included 158 candidates. Approximately 20 of those preparations were in an advanced stage of development, including mRNA-based vaccines, adenovirus-based vaccines and pathogen-specific vaccines [5]. During the summer of 2020, experimental vaccines exhibiting good results in clinical testing were based on inactivated or live attenuated viruses, protein subunits, virus-like particles, viral vectors (either replicating or nonreplicating), DNA and mRNA delivered by chemically synthesized nanoparticles (NPs; i.e., liposomes) [6]. Of the vaccines racing for approval in mid-2020, only a few obtained FDA/EMA authorizations for emergency use. The first mRNA-based vaccine (Pfizer-BioNTech) was authorized for emergency use by the FDA [7] and EMA [8], followed by the Moderna vaccine on 18 December 2020 [9], and on the 23 August 2021, Pfizer-BioNTech received definitive authorization [10]. At the time of this writing, 21 vaccines have been approved/authorized worldwide and many more are in clinical trials according to the Regulatory Affairs Professional Society website [11]. In addition to Comirnaty (BNT162b2), Moderna COVID-19 vaccine (mRNA-1273), COVID-19 vaccine AstraZeneca (AZD1222), and COVID-19 vaccine Janssen (JNJ-78436735), which are approved in an emergency or definitive use fashion, the list of approved vaccines comprises Sputnik V, Sputnik Light, CoronaVac, BBIBP-CorV, EpiVacCorona, Convidicea, Covaxin, WIBP-CorV, CoviVac, ZF2001, QazVac, COVIran Barekat, Abdala (CIGB 66), Soberana 02 and three more still-unnamed vaccines approved in China and Iran [11].

In general, vaccination increases people's health and subsequent survival and protects populations and communities from new and/or re-emerging health threats, enhancing societal productivity. The seminal question for a successful vaccination campaign to stop a pandemic viral infection is how to establish efficient vaccines that can be quickly available for the entire world population. Indeed, vaccination requires not only an efficient vaccine but also research for the timely improvement of the formula for the expected evolution of viral genetic variants. Innovative, effective, safe and affordable preparations are critical to achieving this goal. Proper financing and health system programs are also needed, as well as credible policy recommendations to enhance public knowledge of the social value of vaccines.

The history of documented vaccines and vaccination spans more than 200 years, as presented in Table 1. The panel of developed vaccines and their histories are intricate. A few examples from the 19th, 20th and 21st centuries are provided to show the different approaches and implementation of molecular features.

**Table 1.** Evolution of vaccines against pathogens in terms of technological development.

Technology	18th century	19th century	20th century	21st century
Genetically engineered vaccine	–	–	Hepatitis B, Lyme, cholera	Human papilloma meningococcal group B, SARS-CoV-2
Purified proteins, polysaccharide vaccine	–	–	Diphtheria toxoid, tetanus toxoid, anthrax, meningococcus polysaccharide, pneumococcus polysaccharide, Haemophilus influenza, typhoid polysaccharide. pertussis, hepatitis B	Pneumococcal conjugate (hepta and polyvalent), meningococcal conjugate
Killed whole organisms vaccine	–	Typhoid, cholera, plague	Pertussis, influenza, rickettsia, polio (injected), rabies, Japanese encephalitis, tick-borne encephalitis hepatitis A, meningococcal group C, cholera	Japanese encephalitis, cholera
Live attenuated vaccine	Smallpox	Rabies	Tuberculosis, yellow fever, polio (oral), measles, mumps, rubella adenovirus, typhoid, varicella, rotavirus, cholera, influenza	New rotavirus, zoster

Edward Jenner began the era of vaccination with an experiment done more than 200 years ago, leading to the first documented vaccine for smallpox, which replaced variolation [12]. In 1979, the 33rd World Health Assembly declared smallpox eradicated. A vaccine for rabies was developed in 1885 by Louis Pasteur, then vaccines for cholera and typhoid were developed in 1896 and a plague vaccine subsequently followed in 1897 [13]. Several other important vaccines were prepared at the beginning of the 20th century, such as the first diphtheria vaccine (1913, Emil Adolf von Behring and William Hallock Park), a whole-cell pertussis vaccine (1914), tetanus vaccine (1927) and yellow fever vaccine (1936, Max Theiler). In 1945, the earliest flu vaccine was approved in the USA [14] and the Salk inactivated polio vaccine (IPV) was introduced in 1955, further replaced by the live and orally delivered Sabin polio vaccine (OPV) in 1962. Due to a comprehensive antipolio vaccination campaign, the WHO declared polio entirely eliminated from the western hemisphere in 1994 [15].

The first live measles vaccine was licensed in 1963 and the Global Vaccine Action Plan, implementing measles vaccination worldwide, led to an 84% decrease in measles deaths since 2000. Unexpectedly, 4 years later, Dr Andrew Wakefield published a report in *The Lancet* describing a link between the measles–mumps–rubella (MMR) vaccine and autism [16]. The indication is aimed at thimerosal, an antiseptic and antifungal organomercury compound used as a preservative in the vaccine. Thimerosal was removed from most vaccines and, together with various other assertions, contributed to beginning the modern antivaccine movement. An important milestone in vaccination was reached when a specific vaccine against the oncogenic human papillomavirus (HPV) commercially known as Gardasil, was approved in 2006, followed by another in 2009 [17]. In 2020, a populational study performed in Sweden showed that among girls and women (ages 10–30 years), quadrivalent HPV vaccination was correlated with a substantially reduced risk of invasive cervical cancer [18].

Critical vaccination challenges emerge from recurrent flu pandemics. Although efficient quadrivalent vaccines against the influenza virus were approved in the 2013–2014 flu season [19], the infection remains a major public health concern. The effectiveness of global vaccination against flu is reduced due to fast viral variability, so the need for vaccines to match the circulating strains is high, in addition to the continuous production of adjuvants and vaccine active molecules. Although 2020 has diverted scientific attention from influenza vaccination, lessons can be learned from the flu vaccination and adapted to the SARS-CoV-2 pandemic [20]. Since the various vaccines were introduced, the cases of those pathologies decreased by 99–100% for most infections. As shown in Table 1, vaccines have evolved from live attenuated viruses to more sophisticated nanosized biotools, like purified molecular antigens or genetically engineered molecules currently in use.

## DNA/RNA-based vaccines & COVID-19-aimed carriers

Designing a vaccine against SARS-CoV-2 infection represents a groundbreaking approach meant to stop viral transmission and acquire herd immunity. From an educational point of view, vaccine platforms can be classified as conventional formulations using manipulated viral particles (e.g., Astra Zeneca and Johnson & Johnson), and innovative ones based on mRNA technology and synthesized delivery systems (e.g., Pfizer and Moderna). The essential recipe for a vaccine comprises the active constituent, which could be the specific antigen (protein or polysaccharide) or a sequence of genetic material (DNA, RNA) able to harbor information to produce the antigen in host cells. Both are frequently administered together with an adjuvant to facilitate the developed innate immune response. Both components are usually embedded in a carrier that protects the active ingredient and properly delivers it to the host. The delivered or produced antigen is intended to generate a specific adaptive immune response leading to immunization and generation of immune memory against the aggressor pathogen [21].

The notion of carriers demands a brief clarification, since in the vaccine development pipeline, ‘carrier' refers to a component aimed at appropriately delivering the key ingredients (e.g., mRNA sequence). This notion should not be confused with the concept commonly used in immunology where the carrier represents a protein linked to an antigen (usually of polysaccharide origin) to achieve a full antibody-mediated response. In this case, the linked protein facilitates the recruitment of T helper (Th) lymphocytes. In particular, the follicular-T helper (T_FH_) cells allow the creation of a lymphoid organ-germinal center, where B lymphocytes undergo somatic mutation, antibody affinity maturation and isotype switching [22,23]. This carrier-conjugate transforms the T cell-independent antigens (e.g., earlier polysaccharide vaccines), into the much more immunogenic T cell-dependent antigenic vaccines [24].

On the other hand, in pharmacology and vaccinology, this concept is associated with an excipient function chaperoning the vaccine active component through the inoculation route to the final target. These excipients require thorough attention in vaccine design, as some of their intrinsic features can make them potential inducers of adverse host responses. Therefore, there is special emphasis on studying the potential excipient effects when the end products are intended for widespread vaccine formulations, especially in the platforms authorized for emergency use against COVID-19 [25].

Traditional vaccine categories were based on the entire virus. Indeed, the forerunners in the field used living pathogens with reduced virulence for vaccinations, for example, those developed against smallpox [26]. The next category comprises inactivated pathogens, often requiring repeated inoculations to accomplish robust and long-term immunity. Experience gathered from long-lasting medical applications has shown that such direct pathogen formulas register a poor safety record, and the manufacturing process can be a source of disease outbreaks in itself [27]. These considerations highlight the necessity for developing vaccines with improved safety and efficacy [28].

The new vaccination era erupted in the early 1990s when the expression of a transgenic protein in a mice model inoculated with plasmids harboring the cloned protein of interest was reported [29]. Denominated as a DNA-based technique, this approach was followed by the employment of viral vectors (i.e., adenoviral vectors) and more recently by RNA vaccine technology. The outstanding point of this innovatory research is that a specific viral protein, or a part of it, is sufficient to induce an effective and specific immunity without involving the entire pathogen particle. The singular moiety-induced, tailored and efficient immune response represents the central theme of groundbreaking RNA-based vaccine research [30]. mRNA-based vaccines, currently taking center stage in the COVID-19 vaccination strategy, are novel, powerful weapons considered very promising against many viral diseases and cancer. These molecular-based vaccines are superior in terms of safety, efficacy and large-scale manufacturing compared with other vaccines. A tremendous amount of work has been performed to cope with high mRNA immunogenicity and instability in order to obtain a suitable formulation keeping the required vaccine efficacy. Moreover, the optimization to provide a safe profile (e.g., lacking antibody-dependent enhancement effect) and the understanding of carrier-based mRNA vaccine dynamics will elucidate the details of action and efficacy in different people's genetic backgrounds [31].

Four highly effective vaccines against COVID-19 are currently being administered in different population groups with emergency use authorization (EUA). Pfizer-BioNTech (BNT162b2), Moderna (mRNA-1273), AstraZeneca (ChAdOx1-S [recombinant]) and Johnson & Johnson (Ad26.COV2.S) are currently being used for large scale vaccination in several countries. The coronavirus vaccines from Pfizer, Moderna and AstraZeneca are given as two-dose, while only one dose of the Johnson & Johnson vaccine is required. Discussion of a further administration (i.e., a third dose of Pfizer or second dose of J&J) is currently ongoing in the effort to quench the spread of the recently developed SARS-CoV-2 genetic mutations. All formulas work by stimulating host cells to produce the SARS-CoV-2 spike protein so the immune system learns to recognize this pathogen-related tag and quickly reacts to the viral infection in real-life exposure [32].

### Traditional platforms in COVID-19 vaccination

The traditional vaccine carriers based on viral vectors use a consolidated technology that employs genetic modification of the original virus to express heterologous proteins of the vaccine target virus. One important issue limiting vaccine efficiency is the presence of specific preexisting antibodies in the host against the carrier virus. For example, using widespread adenoviruses (Ad) as carriers could limit vaccination efficacy due to the frequent presence of anti-Ad antibodies raised against different proteins of the capsid. This problem can be surmounted by optimizing the vaccine key antigen (S or N protein) so that the preexisting Ad-antibodies do not represent an obstacle to vaccine effectiveness [33]. Oxford University and AstraZeneca developed a vaccine based on a recombinant chimpanzee adenovirus (ChAd) ChAdOx1-nCoV-19 (recently commercially renamed Vaxzevria), comprising the gene that encodes for an optimized codon of the SARS-CoV-2 S protein. In a mouse model, this vaccine induced vigorous humoral and Th-1 cell-mediated response, while studies in Rhesus macaques revealed an immune response from both Th-1 and Th-2 lymphocytes. In this model, the T-mediated immune response correlated with a reduced viral load in animals exposed to SARS-CoV-2 [34,35]. Research aimed at understanding the efficacy of ChAdOs1-nCoV-19 on the original virus and the rising variants is ongoing, analyzing both trial data [36] and actual clinical results [37].

Another in-use viral vector-based vaccine against COVID-19 currently under EMA investigation is the Sputnik-V developed at Gamaleya Research Institute of Epidemiology and Microbiology in Russia [38]. The formula comprises a combination of two human adenoviruses, Ad26 and Ad5, containing the full-length S gene that encodes for the antigen of interest [39]. Ad26 is injected in the first dose, whereas Ad5 is used to boost the immune response as a second administration. As well as the other SARS-CoV-2 vaccines, Sputnik-V safety and efficiency are currently under investigation by analysis of randomized controlled trials [39]. All this data analysis, together with the knowledge coming from the vaccination campaigns in the different countries, will clarify the real performance of adenovirus-based carrier vaccines as well as their potential adverse effects, briefly reviewed in a following section.

### Approved/authorized & applied innovative vaccine platforms

The Pfizer vaccine is based on a 5′-capped mRNA single-stranded molecule that encodes the spike protein S of SARS-CoV-2, an essential protein for virus intrusion into the host cells. The mRNA sequence is entrenched in lipid nanoparticles (LNPs), a nano-delivery system of mRNA vaccines that has shown encouraging results in Zika and influenza epidemics [40,41]. Similar to the Pfizer vaccine, the Moderna (mRNA-1273) formula contains a synthetic mRNA molecule encoding for SARS-CoV-2 spike protein formulated in LNPs (https://www.fda.gov/media/144434). Along with polyethylene glycol (PEG) 2000, Moderna includes as excipient tromethamine that can potentially lead to immune hypersensitivity reactions (IHRs) [42]. Pfizer-BioNTech (BNT162b2) and Moderna (mRNA-1273) are framed in PEGylated liposomes. These synthetic phospholipid vesicles have proven to be efficacious as molecule stabilizers and uphold pharmacologic properties. Especially in the case of the COVID-19 vaccine, the instability of mRNA molecules demands such preservations in LNP-based formulations [43]. The patrolling innate immune cells quickly recognize and process the translated spike 2 protein. These mRNA-based vaccines reach roughly 95% protection from COVID-19 eliciting both humoral and Th1-mediated responses. Although, the precise time that the immunizations last is still unclear, and further data collection from ongoing worldwide vaccination are needed [44,45].

### Adverse immune reactions to the carrier

Vaxzevria (ChAdOx1-nCoV-19) has been largely used in the UK, where more than 30 million people received at least a first dose of the vaccine. However, critical issues regarding its safety have been raised by different EU countries, temporarily stopping its administration. The EMA is monitoring the eventual cause–effect relationship with rare unusual thrombosis and thrombocytopenia events that occurred upon vaccination [46,47]. Potential molecular mechanisms of this adverse event are emerging from postmarketing research [48]. Antibodies against PF4 have been found in 95% of a small group of patients showing acute thrombocytopenia and thrombosis 6–24 days after receiving the first dose of the AstraZeneca vaccine. The described adverse events are extremely rare. Very recent medical treatments based on immediate therapy with nonheparin anticoagulants and high doses of intravenous immunoglobulins (IVIGs) successfully helped patients recovery. In particular, IVIGs competitively inhibit the platelet-activating effects of ChAdOx1nCoV-19 vaccine-induced antibodies [49].

Another potential cause of IHRs developed after AstraZeneca vaccination is represented by PS80, a surfactant with a polymeric structure similar to PEG, used in many injectable preparations [50]. PEGylation is a common coating strategy to impede the vaccine carrier aggregation at the inoculation site, also used for the Pfizer and Moderna delivery systems. The PEGylated liposomes carrying the SARS-CoV-2 spike protein-encoding mRNA have recently raised concerns for potential anaphylaxis, especially if multiple injections are required in the future [51]. These coating polymers favor the carrier spreading to regional lymph nodes without hindering recognition and supporting the uptake of vaccine NPs by antigen-presenting cells (APC) in the first h after inoculation.

As mentioned, PEG coating is crucial for the stability of liposomal vaccine formulations but some general concerns regarding its safety are emerging, as the presence of anti-PEG antibodies has been reported [52–55]. Specifically, the analysis of blood samples from patients treated with PEG-conjugated drugs presented detectable levels of anti-PEG IgM and IgG [53]. The generation of anti-PEG antibodies in response to recurrent PEG dose administrations can lead to accelerated blood clearance (ABC) of the PEGylated drug, reducing its circulation time. Either IHR or complement activation-related pseudo-allergy (CARPA) may be induced by anti-PEG IgE [56,57].

The choice of the optimal vaccine platform for frail patients with rare diseases is even more complex. In patients diagnosed with mastocytosis, a rare disease caused by excessive mast cell accumulation in different organs, an increased risk of anaphylaxis has been reported [58]. A rapid chain of events after vaccination could increase the probability of anaphylaxis in these patients. The release of anaphylaxis mediators is accomplished mainly through IgE cross-linking induced by LNPs. Moreover, as a result of LNP-mediated tissue damage, the released IL-33 can bind to the orphan receptor ST2 on IgE-sensitized mast cells, thus sustaining the anaphylaxis [59]. Vaccination campaigns in the UK, Canada and the USA, have reported some anaphylactic reactions. Further investigations suggested the excipient PEG-2000 used in both Pfizer and Moderna vaccines as the potential allergen. Such allergic reactions can be IgE-mediated but may also result from CARPA developed by analogous liposomes. As mentioned, Moderna also contains tromethamine/trometamol, a potential contributor to anaphylactic reactions [42,60].

## Vaccine carriers in immune milieu vaccination

The plethora of data extracted from humoral and cell-mediated immune responses against pathogens has fueled research into novel particulate vaccines design. Polymer-based nanostructures have been integrated as part of such innovative endeavors, especially for nucleic acid-based strategies. These polymer-based nanostructures are particularly prone to functionalization chemistry with good biocompatibility. Their structure can be tailored to function as real biocontainers, storing, delivering and releasing the antigen of interest for protective immunity [61,62]. The immunological basis for particulate vaccine delivery is a key feature that dictates the vaccination-induced long-term protection against the infectious agent. Therefore, many carriers are designed to target lymph nodes (LN) and trigger a specific immune response by delivering their cargo. LNs are the prime stations where immune activities start, a seminal site in which a network of APCs, B and T lymphocytes cooperate through a multitude of receptors/signals to develop a specific immune response tailored to the invader's features. The first defense against a pathogen takes place at mucosal surfaces or the skin being interceded by the complement system and specialized APCs, macrophages and dendritic cells (DCs). The following immune cascade is continued by the acquired (or adaptive) immune arm comprising B and T lymphocytes that build up the long-term immune memory, assuring a rapid protective reaction when the host reencounters the same antigen [63,64].

As previously reported, Pfizer and Moderna have lipid NP formulations containing mRNA encoding for the S protein of SARS-CoV-2, whereas AstraZeneca and Sputnik are double-stranded DNA adenovirus (AdV)-based vaccines encoding the same S protein. These formulations are injected into the muscle where they are captured by DCs at the injection site and further distributed through the lymphatic system into different LN's cellular areas. The result is the production of high levels of S protein enemy-tag that will instruct the host immune cells to remember the aggressor antigen.

**Figure 1.**
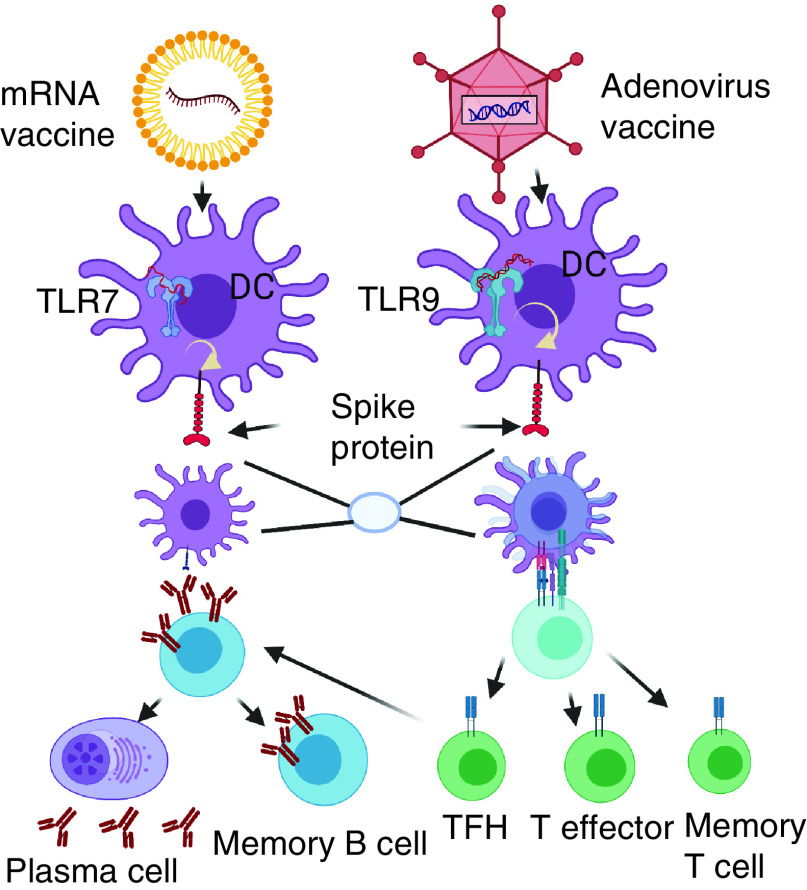
Immune pathways triggered by the two main approved vaccines: mRNA-type and adenovirus vaccine. Both vaccine types enter into resident dendritic cells at the injection site. mRNA type uses TLR7 as a biosensor and induce protein spike synthesis and expression by passing the nucleus machinery; adenovirus vaccines use TLR9 as a biosensor and induces protein spike synthesis and expression using the nucleus machinery. TLR7 and TLR 9 induce the production of Type I interferon and multiple proinflammatory cytokines/chemokines that activate dendritic cells. Activated dendritic cells will present antigen and costimulatory molecules to specific naive T cells, which become activated and further differentiate into effector cells (T cytotoxic cells), T helper cells and memory T cells. T follicular helper cells help B cells differentiate into plasma cells that will secrete specific antibodies and B memory cells. T and B memory cells will sustain long-lasting memory of the infection and the second encounter with the same virus will quickly trigger the necessary immune pathways. Figure created with Biorender.com. DC: Dendritic cell.

Concomitantly, the spike single-strand mRNA of the Pfizer and Moderna vaccines is detected by specific innate immune cell receptors, such as TLR7 and MDA5. On the other hand, the double-stranded DNA within the AdV vaccines is detected by other TLRs, such as the TLR9, which is a major sensor for double-strand DNA. The common result of the vaccine-embedded genetic strings is a significant production of several proinflammatory cytokines and chemokines [65]. Per se AdV vaccines have inherent adjuvant properties, residing within the virus NP that encases the DNA encoding the immunogen. Following injection, AdV will activate multiple pattern-recognition receptors that bind double-stranded DNA, in particular TLR9 that will generate the entire immune cascade with the final scope to express the foreign immunogen [66]. mRNA vaccines do not activate TLR9, but single-stranded RNA (ssRNA) will be recognized by TLR3 and TLR7 in the endosome, and components of the inflammasome (e.g., MDA5, RIG-I, NOD2 and PKR) will bind to ssRNA and dsRNA in the cytosol, resulting in overall cellular activation, including the production of IFNI and other inflammatory mediators [67]. Outlines of the mRNA-based vaccines and AdV vaccines immune pathways is presented in Figure 1.

Nanovesicles are engulfed by DCs such that the antigenic cargoes carried in the polymeric particles can be digested into smaller antigenic peptide fragments that are further presented on the surfaces of DCs in association with the MHCs system [68]. Antigen delivery and processing via the MHC II class pathway are influenced by the size, shape and charge of the polymeric particles [69]. A variety of antigens can be encapsulated, including antigen-encoding nucleic acids (RNA or DNA), as is the case for anti-COVID-19 vaccination platforms [63]. After vesicle engulfment, this primed complex formed by activated DCs and costimulatory molecules (CD28, CD80/CD86) will present the peptide-MHC II complex to the T-cell receptor (TCR) expressed by S protein-specific naive T cells. As a result, naive T cells become activated and differentiate into effector (cytotoxic) T CD8^+^ lymphocytes (CTL) or CD4^+^ Th. The further differentiation of naive CD4^+^ T cells into different subsets is also activated by their interaction with DCs in lymphoid organs and modulated by various cytokines that play a key role during the early phases of the process [70].

The comprehension of the cellular response to the COVID-19 vaccine will require profound analysis of millions of cases. The peptide process by APCs to T-lymphocytes is defined by intricate and overlapping cellular communications leading to the optimal host reaction. Ongoing research has shown that depending on the immune response requirements, Th cells can differentiate into conventional Th1 and Th2, along with Th17 cells, T_FH_ cells, induced T regulatory cells (iTreg), regulatory type 1 cells (Tr1) as well as the Th9 subset. Each of these T lineages encompasses a complex network of transcription factors and specific cytokine patterns and are accompanied by epigenetic alterations [71–73]. Th1 cells can produce IFN-γ and IL-2 to activate CTL that promotes cell-mediated immunity. In addition, the Th1 subtype contributes to the development of long-term memory T lymphocytes. Along with Th1, the Th2 subpopulation supports humoral immunity development by releasing immunoregulatory cytokines, such as IL-4, IL-5, IL-10 and IL-13, that have also been shown to exhibit anti-inflammatory actions on various cellular types. Furthermore, the Th2 subtype activates B cells to secrete specific antibodies against antigens usually linked to extracellular pathogens [74,75]. The Th17 subpopulation secretes cytokines from the IL-17 family to promote inflammation as a response to infections. Treg cells are another important subpopulation in preserving immune tolerance and regulating the extent of immune responses by controlling the differentiation and functions of T effector cells [76].

T_FH_ cells mediate humoral immunity via B lymphocytes interaction in the germinal center and assist B cells in differentiating in plasma cells that subsequently produce high-affinity antibodies. T_FH_ cells assist B cells to differentiate into memory B cells to sustain long-lasting immune protection. In particular, for the COVID-19 vaccine nanovesicle, the T_FH_ subset assists S protein-specific B lymphocytes to differentiate into antibody-secreting plasmocytes and endorse the production of high-affinity anti-S protein antibodies [65,76]. The early innate immune response also contributes to driving the fate of the cellular adaptive response due to the costimuli of the adjuvants in the vaccine preparation. Immune adjuvants can be divided into immune enhancers (e.g., mineral salts) and vaccine-delivery platforms (e.g., liposomes). Liposomes, as innovative carriers in the mRNA vaccine concept, fall in the category of particulate-delivery systems along with microsphere polymers or virus-like particles [77–79]. Embedding mRNA in a lipid capsule also favors the internalization of the immune inducer into cytosol, preparing it for translation and preventing potential inflammatory/toxicity processes provoked by naked mRNA [80]. Upon vaccination, it is expected that S protein-specific memory T and B lymphocytes will develop and start to patrol along the blood–lymph–lymph nodes route, accompanied in the circulation by high-affinity SARS-CoV-2 antibodies [81]. In this manner, this specific immune army establishes a protective battery to prevent and fight any subsequent infection with SARS-CoV-2 [64]. However, the comprehension of the specific role, functions and fate of these B and T lymphocytes populations in the COVID-19 immunization setting has yet to be revealed and is currently a matter of tremendous scientific interest and effort.

New systems broaden the vaccine carrier domain, such as the one published in April 2021 regarding the development of cell-free gene expression (CFE) systems with cell-derived membrane-dependent functions. In this platform, membrane-bound cargo expressed in live *Escherichia coli* comprises membrane vesicles. CFE nano-characterization pipeline can be a promising platform for vaccine development [82]. All current and future nano-carriers that transport the different active compounds must display delivery efficiency, cell targeting, materials safety and thermostability [82–84].

## Conclusion

The two-century-long path of vaccine development has enormously improved human life. Vaccine applications started with rough empirical applications for a life-or-death choice and have evolved to display modern formulations that obey safety rules and regulations until mass vaccination approval. The continuous understanding of the pathological mechanisms and the achievement of sophisticated molecular biology techniques, as well as the progress made in the biomaterial domain, have facilitated the battle against microorganisms. However, the dogma: ‘*the more you know the more you have to learn’* takes on a clear meaning with modern spreading pandemics. Notwithstanding nanotech-vaccine availability, viruses will always challenge human strength and skills. To cope with the future pandemic virus variants that will appear as their biological evolution will proceed, we must rely on solid vaccine research for globally available precision medicine.

## Future perspective

As most viral vaccines primarily aim to generate an antibody-mediated immune response, the near future in COVID-19 vaccination will require important information regarding T and B cell immunity in COVID-19 patients and in vaccinated subjects. These studies can guide the development of strategies for effective protection from SARS-CoV-2. As SARS-CoV-2 and its various variants will likely circulate for many years to come, efficient and safe COVID-19 vaccines should be developed for anticipated viral mutations. Thus, new formulations extending the administration routes and vaccination timing should be pursued. New formulations may decrease side effects, and nano-sized materials from natural sources will enter the pipeline. The speed with which the mRNA-based vaccines were developed may lead to a new era in other domains, such as oncology.

**Executive summary**

**Background**

- In the case of viral infections, the most effective way to obtain herd immunity is the development of efficient and safe vaccines.
- The SARS-CoV-2 pandemic generated the development of various vaccine platforms, including mRNA-based vaccines, adenoviruse-based vaccines and pathogen-specific vaccines.
- The history of documented vaccines and vaccination spans more than 200 years, culminating with currently available genetically engineered vaccines.

**DNA/RNA based vaccines & COVID-19-aimed carriers**

- The design of a vaccine comprises the active constituent, for example, an antigen (protein or polysaccharide) or a sequence of genetic material (DNA, RNA) able to harbor and further produce the antigen in host cells and an adjuvant that facilitates the development of the immune response.
- Vaccine platforms can have conventional formulations using manipulated viral particles or innovative ones based on mRNA technology.
- The traditional vaccine carriers based on viral vectors use genetic modification of the original virus to express heterologous proteins of the vaccine target virus.
- Innovative vaccine platforms comprise a 5′-capped mRNA single-stranded molecule that encodes the spike protein S of SARS-CoV-2.
- The mRNA sequence is entrenched in lipid nanoparticles, a nano-delivery system with PEG2000 or added excipients, such as tromethamine, components that can potentially induce immune hypersensitivity reactions.

**Vaccine carriers in immune milieu vaccination**

- The immunological basis for the particulate vaccine delivery is a key feature for the events induced by the vaccination and long-term protection against an infectious agent.
- Intimate cellular immune mechanisms triggered by mRNA or DNA-based vaccines are different.
- The spike's single-strand mRNA is detected by specific innate immune cell receptors, such as TLR7 and inflammasomes components, like MDA5.
- Double-stranded DNA within the adenovirus vaccines is detected by innate immune cell receptors, TLR9, the main sensor for double-strand DNA.
- Whether mRNA or DNA-based, the further immune route is identical, namely, S protein-specific memory T and B lymphocytes will be developed, accompanied in the circulation by specific high-affinity antibodies.

**Conclusion**

- To cope with future virus variants, solid vaccine research is needed for globally available precision medicine.

**Future perspective**

- New formulations extending the administration routes and vaccination timing will be pursued, likely reducing the induced side effects of nano-sized materials.
- The research speed registered for the mRNA-based vaccines will encourage a new era of vaccination in oncology.
